# The functional effects of a dominant consumer are altered following the loss of a dominant producer

**DOI:** 10.1002/ece3.10342

**Published:** 2023-08-02

**Authors:** Samuel A. Mahanes, Cascade J. B. Sorte, Matthew E. S. Bracken

**Affiliations:** ^1^ Department of Ecology and Evolutionary Biology University of California Irvine California USA

**Keywords:** algae, dominant species, ecosystem function, intertidal, multifunctionality, mussel

## Abstract

Human impacts on ecosystems are resulting in unprecedented rates of biodiversity loss worldwide. The loss of species results in the loss of the multiple roles that each species plays or functions (i.e., “ecosystem multifunctionality”) that it provides. A more comprehensive understanding of the effects of species on ecosystem multifunctionality is necessary for assessing the ecological impacts of species loss. We studied the effects of two dominant intertidal species, a primary producer (the seaweed *Neorhodomela oregona*) and a consumer (the shellfish *Mytilus trossulus*), on 12 ecosystem functions in a coastal ecosystem, both in undisturbed tide pools and following the removal of the dominant producer. We modified analytical methods used in biodiversity–multifunctionality studies to investigate the potential effects of individual dominant species on ecosystem function. The effects of the two dominant species from different trophic levels tended to differ in directionality (+/−) consistently (92% of the time) across the 12 individual functions considered. Using averaging and multiple threshold approaches, we found that the dominant consumer—but not the dominant producer—was associated with ecosystem multifunctionality. Additionally, the relationship between abundance and multifunctionality differed depending on whether the dominant producer was present, with a negative relationship between the dominant consumer and ecosystem function with the dominant producer present compared to a non‐significant, positive trend where the producer had been removed. Our findings suggest that interactions among dominant species can drive ecosystem function. The results of this study highlight the utility of methods previously used in biodiversity‐focused research for studying functional contributions of individual species, as well as the importance of species abundance and identity in driving ecosystem multifunctionality, in the context of species loss.

## Graphical abstract

We studied the effects of two dominant intertidal species, a primary producer (the seaweed *Neorhodomela oregona*) and a consumer (the shellfish *Mytilus trossulus*), on 12 ecosystem functions in a coastal ecosystem, both in undisturbed tide pools and following the removal of the dominant producer. We found that the dominant consumer—but not the dominant producer—was associated with ecosystem multifunctionality, and the relationship differed depending on whether the dominant producer was present. Our findings suggest that interactions among dominant species can drive overall ecosystem function.

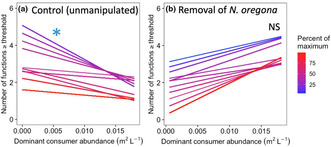

## 1 INTRODUCTION

Global change is driving biodiversity loss worldwide, making it more important than ever to understand the different roles that individual species play in ecosystems (Bellard et al., 2012; Mantyka‐Pringle et al., 2012; Valiente‐Banuet et al., 2015). Whereas most previous biodiversity research focused on the effects of species loss on one ecosystem function (e.g., productivity; Cardinale et al., 2007), it is important to recognize that species simultaneously mediate multiple functions (Gamfeldt et al., 2008; Hector & Bagchi, 2007). Quantifying the role of a species in an ecosystem—and understanding the functional consequences of loss—requires evaluating that species' simultaneous contributions to multiple ecosystem functions (e.g., net primary productivity, decomposition, nutrient cycling), also known as “ecosystem multifunctionality” (Manning et al., 2018).

Much of the multifunctionality research conducted to date has focused on the effect of community‐level biodiversity on ecosystem functions (Tolkkinen et al., 2013). Community diversity has been shown to strongly influence ecological function, both at the scale of single functions and overall multifunctionality within an ecosystem (Hector & Bagchi, 2007; Zavaleta et al., 2010). Researchers have identified a combination of sampling and species identity effects, by which individual species, rather than the number of species per se, are the primary drivers of the biodiversity–multifunctionality relationship (Brun et al., 2022; Cardinale et al., 2006; Slade et al., 2017). Individual species, particularly those that are highly abundant in an ecosystem, have emerged as potential key drivers of ecosystem multifunctionality (Fields & Silbiger, 2022; Hillebrand et al., 2008; Lohbeck et al., 2016). Applying methodologies designed for biodiversity–multifunctionality studies (Byrnes et al., 2014) may allow us to further elucidate the functional effects of numerically dominant species.

Dominant species may serve as primary drivers of ecosystem function or, if they are weak functional contributors, potentially limit ecosystem multifunctionality (Hillebrand et al., 2008; Orwin et al., 2014; Wohlgemuth et al., 2016). Dominant species, defined based on their abundance (e.g., >12% relative abundance in community; Mariotte et al., 2015), display a wide variety of forms across ecosystems, from the northern red oak (*Quercus rubra*) in the forests of the northeastern United States (Ellison et al., 2019) to red oat grass (*Themeda triandra*) in the shrublands of South Africa (Cowling, 1983). The more abundant a species is in an ecosystem, the more likely it is to significantly influence local environmental conditions and overall ecosystem function (Brun et al., 2022; Ellison, 2019; Lohbeck et al., 2016; Tolkkinen et al., 2013; Wohlgemuth et al., 2016). This phenomenon is typified by the “mass ratio hypothesis,” which states that the functional traits of dominant species in an ecosystem will strongly influence ecosystem processes (Grime, 1998; Orwin et al., 2014). Understanding how dominant species contribute to ecosystem function, as well as the possibility that they limit overall ecosystem function by crowding out other species (Altieri et al., 2009; Tingley et al., 2002), is critical for understanding how climate change and biodiversity loss will impact ecological function (Giling et al., 2019; Hillebrand et al., 2008; Tolkkinen et al., 2013).

Many ecosystems contain multiple dominant, foundation, and/or habitat‐forming species, and the interactions between these species may affect ecosystem functioning (Angelini et al., 2011; Austin et al., 2021). Altieri et al. (2007) documented interactions between dominant species on cobble‐beaches: where cordgrass aggregations and ribbed mussel beds overlap, they interact to produce a shaded, wave‐sheltered habitat that supports higher species diversity than the surrounding area. The functional complementarity of some pairs of dominant species, as well as the potential facilitation of one dominant species by another (Angelini et al., 2011), raises the question of how an ecosystem would be affected by the loss of one of multiple dominant species present (Angelini & Silliman, 2014). If the dominant species compete (e.g., for space; Yakovis et al., 2008), have a facilitative relationship (e.g., through complementary nutrient cycling; Aquilino et al., 2009), or exert an interactive effect on the ecosystem (e.g., by forming complex habitat; Altieri et al., 2007), the loss of one species may affect the other dominant species and ultimately ecosystem function. In this study, we investigated the contributions of, and potential interactions between, a pair of dominant species—the algal producer *Neorhodomela oregona* and bivalve consumer *Mytilus trossulus*—to critical functions in coastal ecosystems.

Many of the key ecological processes in coastal ecosystems can be grouped into three sets of functions: productivity, nutrient cycling, and effects on water chemistry (Tolkkinen et al., 2013). Primary productivity is the fixation of carbon via photosynthesis and can be measured though oxygen production and related chemical fluxes (Bracken & Williams, 2013). Primary productivity has been strongly associated with the functional traits of dominant species (Bruno et al., 2006; Mouillot et al., 2011), raising the possibility that the association between biodiversity and productivity is predominantly an effect of these abundant, functionally unique species being included more frequently in more biodiverse samples (i.e., sampling effect; Aarssen, 1997; Huston, 1997).

Primary production, itself, can be limited by nutrient availability (Bruno et al., 2006), which positions the cycling of ammonium, nitrate, nitrite, and phosphate as critical to the overall functionality of coastal ecosystems (Bracken & Williams, 2013; Vanni, 2002). While nitrate and phosphate can reach high concentrations in coastal waters, ammonium—which is typically at low concentrations in seawater due to preferential uptake—often accumulates in tide pools, due to excretion by invertebrates (Aquilino et al., 2009; Bracken & Nielsen, 2004; Bracken & Williams, 2013). Local‐scale accumulation of ammonium and phosphate in coastal ecosystems has been directly tied to the abundance of mussels (Asmus et al., 1995; Bracken & Nielsen, 2004), which corroborates findings that nutrient‐limited seaweeds are more abundant and grow more rapidly on mussel beds than on other intertidal surfaces (Aquilino et al., 2009; Bracken, 2004). The dominance of different species in otherwise similar communities can lead to divergence in nutrient cycling rates among communities (Bracken & Williams, 2013; Wohlgemuth et al., 2016). Because seaweeds can account for most of the primary productivity in temperate coastal ecosystems (Mann, 1973) and can strongly influence nutrient fluxes in these ecosystems (Bracken & Nielsen, 2004), understanding the contributions of dominant seaweeds to individual ecosystem functions and ecosystem multifunctionality is critical for anticipating impacts of ongoing species loss.

Dominant species in coastal ecosystems may drive changes in other characteristics of water chemistry, with implications for rates of ocean acidification (Aiuppa et al., 2021; Kroeker et al., 2013). Marine producers can raise seawater pH via photosynthesis (Bracken et al., 2018) as well as increase pH variation over diel cycles, which may help mitigate local‐scale acidification in marine ecosystems (Camp et al., 2016; Wahl et al., 2018). However, producers may also reduce pH in the absence of light, when photosynthesis ceases but respiration continues, shifting the balance from a reduction of inorganic carbon in the water column to a net increase and contributing to further acidification (Krause‐Jensen et al., 2015; Mahanes et al., 2022; Silbiger & Sorte, 2018). Producer‐driven changes in pH can affect other species in the ecosystem, particularly calcifying species (e.g., mussels and oysters; Semesi et al., 2009; Wahl et al., 2018), which are disproportionately impacted because calcification, the process in which organisms absorb calcium carbonate from the water column to build body structures, can be reduced at low pH (Kroeker et al., 2013). Acidification shifts the chemical equilibrium toward calcium carbonate dissolution, raising the metabolic cost of calcification for organisms or preventing calcification altogether (Andersson & Gledhill, 2013); therefore, robustly photosynthetic species can serve an important function by raising seawater pH.

We assessed the effects of dominant species from different trophic levels on individual ecosystem functions, groups of functions, and overall multifunctionality in coastal systems, both when acting in concert and after simulated species loss. We conducted a removal experiment on the dominant algal producer *N. oregona* in tide pools where the mussel *M. trossulus* was also highly abundant, and we subsequently applied a methodology from biodiversity–multifunctionality studies to measurements of 12 ecological functions. Based on the results of past studies on comparable seaweed and mussel species (e.g., Mahanes et al., 2022), we predicted that the dominant producer species would contribute to ecosystem productivity, raise pH, increase calcification, and drive nutrient absorption, while the dominant consumer was expected to increase respiration, reduce pH, increase calcification, and drive nutrient accumulation.

## 2 MATERIALS AND METHODS

### 2.1 Study site

We studied effects of the dominant Oregon pine seaweed (*Neorhodomela oregona* [Doty] Masuda) and Pacific blue mussel (*Mytilus trossulus* Gould) on ecosystem function in a coastal ecosystem. *N. oregona* is a turf‐forming seaweed which is numerically dominant in tide pools at John Brown's Beach on Japonski Island, Sitka, Alaska, USA (57.06° N, 135.37° W), comprising >55% of total tide pool surface area (Mahanes et al., 2022). *N. oregona* is common in tide pools throughout Southeast Alaska, and its range spans the North Pacific from California to parts of Japan and Russia (Lindeberg & Lindstrom, 2016). *M. trossulus* is a sessile mussel species, generally smaller than its relatives *M. californianus* and *M. galloprovincialis*, which can form dense aggregations and is commonly found along the coastline from California to Alaska, USA (Braby & Somero, 2006). *Mytilus trossulus* is a dominant species in tide pools at John Brown's Beach, accounting for >30% of tide pool surface area (Mahanes et al., 2022). The coexistence of these two species provided an opportunity to investigate the effects of and interactions between two numerically dominant species across a set of tide pools which function as individual, largely self‐contained ecosystems when isolated during low tide (Sorte & Bracken, 2015). To quantify the degree to which a dominant producer and a dominant consumer drive ecological function, we conducted a species‐removal experiment at our study site from July 5 to July 19, 2019.

#### 2.1.1 Tide pool physical characteristics

We selected 10 tide pools with similar dimensions and tide height (i.e., position within the intertidal zone) for this study. We measured the physical characteristics of the tide pools by: (1) pumping the water from a tide pool into a graduated bucket to assess volume, (2) placing a flexible mesh quadrat with 10 cm × 10 cm squares on the bottom of each tide pool to measure basal surface area (Bracken & Nielsen, 2004; Silbiger & Sorte, 2018; Sorte & Bracken, 2015), and (3) using a sight level and a surveying rod to gauge tide height in meters (above mean lower‐low water). We assigned experimental treatments to the tide pools by repeatedly randomizing assignments until volume, surface area, tide height, *N. oregona* abundance (calculated as percent cover), and species richness (calculated from community survey data, see below) did not vary between treatments (*N* = 5, removal or control, based on a generalized linear model with threshold of *p* > .2). The abundance of *N. oregona*, *M. trossulus*, and all other species present was assessed via biodiversity surveys following methods used by Bracken and Nielsen (2004; Appendix S1).

### 2.2 Ecosystem function data collection

**FIGURE A1:**
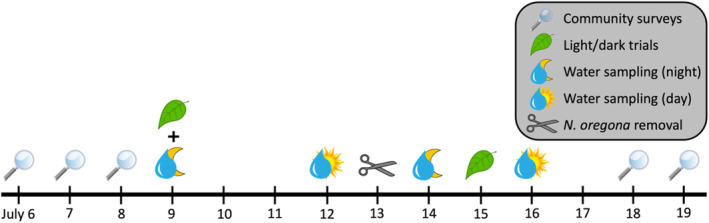
The data used in this project were collected from *N* = 10 tide pools at John Brown's Beach near Sitka, Alaska, USA during a 14‐day time period in July 2019. Water sampling was conducted as close as possible prior to and immediately following the removal of a dominant producer, the alga *Neorhodomela oregona*, to minimize the possibility for uncontrolled factors (such as changing weather patterns) to influence measurements.

We measured the net community productivity and respiration, as well as day and night rates of net ecosystem calcification and pH change, and the fluxes of ammonium, nitrate and nitrite, and phosphate in the experimental tide pools during both day and night. We conducted light/dark productivity trials, as well as time‐series water samplings during the day and night, on the unmanipulated experimental tide pools between July 9 and 12, 2019 (for a timeline of the experiment and sampling, see Figure A1). On July 13, we initiated the manipulations and removed *N. oregona* from the removal treatment tide pools with scissors, cutting as close to the holdfast as possible without damaging any surrounding species. We then repeated the productivity trials and water samplings on the full set of tide pools between July 14 and 16, 2019 (Figure A1).

#### 2.2.1 Light/dark productivity trials

To assess impacts of these dominant species on the productivity of the tide pools, we conducted light/dark incubation experiments before and after the removal of *N. oregona* (Bracken et al., 2022; Noël et al., 2010; Sorte & Bracken, 2015; Figure A1). We took initial dissolved oxygen measurements from each tide pool with a ProDSS Multiparameter Water Quality Meter (YSI). We then covered each pool with an opaque, black tarp for 30 min of dark incubation. We repeated the measurements and then removed the tarps for a 30 min light‐incubation period, at the end of which we took a third and final set of measurements.

#### 2.2.2 Water sample collection

To assess impacts of these dominant species on tide pool water chemistry and nutrient fluxes, we conducted paired time‐series samplings (day and night) before and after *N. oregona* removal (Figure A1). We sampled across three time points over a ~2.5 h time series following isolation of the tide pools from the ocean, collecting water chemistry samples at each time point (Silbiger & Sorte, 2018) by hand‐pumping 250 mL of water from the bottom of the tide pool into a vacuum flask, and then siphoning the water into two 125 mL amber glass sample bottles to minimize gas exchange. We added the remaining water to a 50 mL plastic tube for nutrient analysis. All containers were rinsed three times with seawater before use. We immediately added 60 μL HgCl_2_ to preserve each 125 mL water chemistry sample and then sealed the sample bottles for later pH and total alkalinity analysis. Nutrient samples were stored on ice while in the field and then frozen at −20°C prior to analysis.

At each time point, we also measured salinity and temperature with a ProDSS Multiparameter Water Quality Meter (YSI) and light intensity with a MQ‐210 Underwater Quantum Meter (Apogee) in each pool. Salinity and temperature data were collected for later use in calculating pH values, and light was recorded to document any changes in weather between sampling dates that might affect biological processes. Samples were processed for pH and total alkalinity according to protocols outlined by Dickson et al. (2007) and nutrient concentrations were analyzed using methods of Bracken et al. (2018; Appendix S1).

### 2.3 Data analysis

#### 2.3.1 Calculated metrics

We calculated rates of change (i.e., slopes) for all water chemistry metrics collected over the three‐sample time series, which included pH, ammonium, phosphate, and nitrate + nitrite. We treated day and night rates of change of each function separately because organisms, particularly producers, may affect these factors differently based on the presence or absence of light. We calculated calcification rate using the formula below (Silbiger & Sorte, 2018).

$$\mathrm{NEC}=\left(\mathrm{\Delta TA}\cdot \rho \cdot V\right)/\left(2\cdot \mathrm{SA}\cdot t\right)$$

where ∆TA is the change in total alkalinity between the first and third time points in the sampling (mmol kg^−1^), ρ is the density of seawater (1023 kg m^−3^), *V* is the water volume of the tide pool (m^3^), SA is the bottom surface area of the tide pool (m^2^), and *t* is the time elapsed (h). The 2 is included because a single mole of CaCO_3_ is formed for every two moles of TA.

We used the dissolved oxygen measurements from the light/dark experiments to calculate net community productivity (NCP) and respiration (*R*) in the tide pools according to the formulas below (Noël et al., 2010; Sorte & Bracken, 2015).

$$\mathrm{NCP}=\Delta {\left[O_{2}\right]}_{\text{light}}/\Delta t_{\text{light}}$$

$$R=|\Delta {\left[O_{2}\right]}_{\text{dark}}/\Delta t_{\text{dark}}|$$

In the formulas, ∆[O_2_] is the change in dissolved oxygen concentration (mg O_2_ L^−1^), ∆t indicates change in time, and “dark” and “light” correspond to the covered and uncovered incubation periods, respectively.

#### 2.3.2 Analyses

**FIGURE 1:**
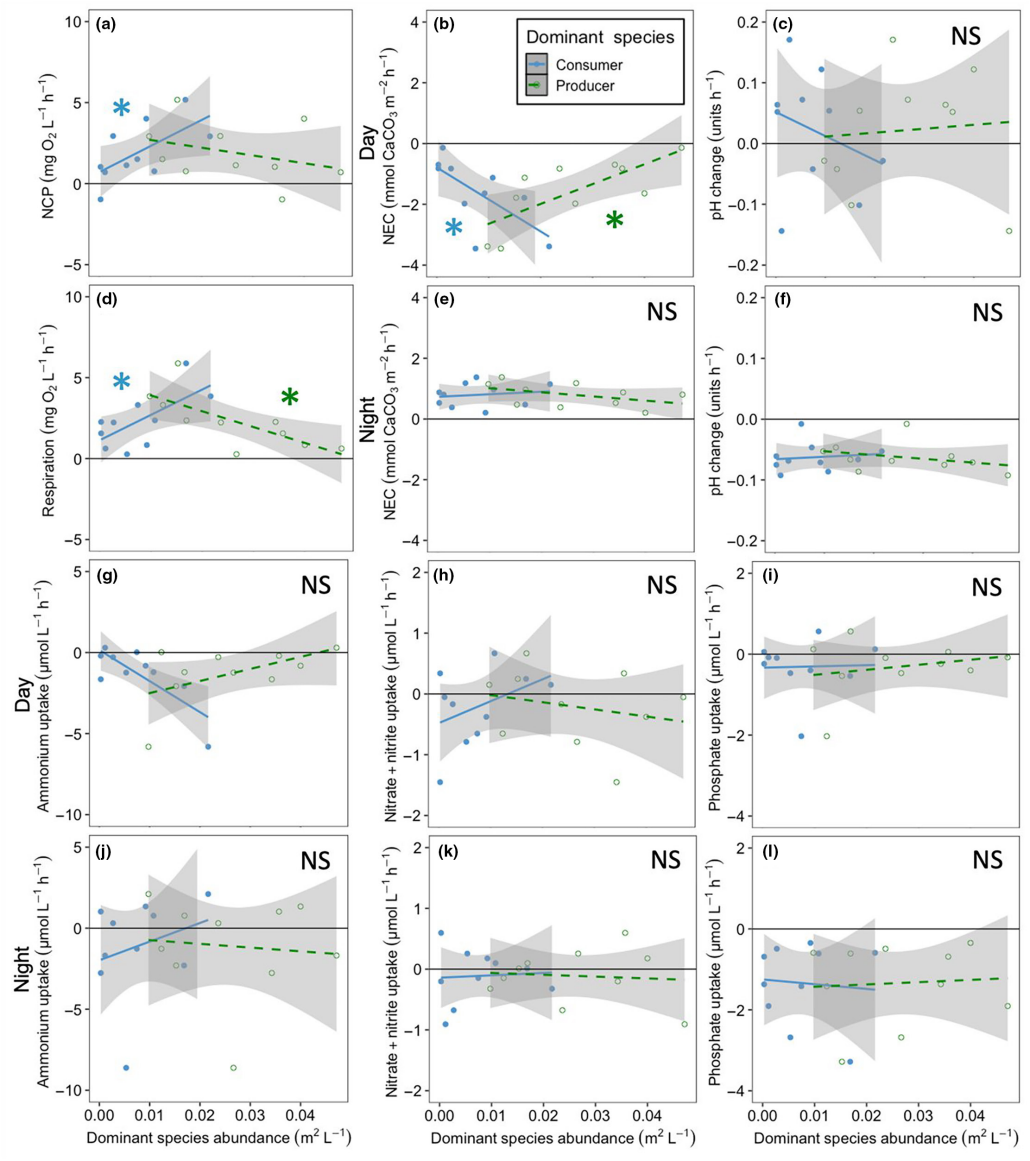
Relationships between the abundances of the dominant producer (the alga *Neorhodomela oregona*; green, open symbols and dashed regression lines) and consumer (the mussel *Mytilus trossulus*; blue, closed symbols and solid regression lines) and 12 individual ecosystem functions: (a) net community production; daytime (b) net ecosystem calcification and (c) pH change; (d) community respiration; nighttime (e) net ecosystem calcification and (f) pH change; daytime (g) ammonium accumulation, (h) nitrate + nitrite uptake, and (i) phosphate uptake; and nighttime (j) ammonium accumulation, (k), nitrate + nitrite uptake, and (l) phosphate uptake. Producer abundance was related to two functions: daytime net ecosystem calcification and respiration. Consumer abundance was related to four functions: net community productivity, daytime net ecosystem calcification, respiration. Each data point represents the abundance of producer (green) or consumer (blue) in a single tide pool. Asterisks indicate significance, NS indicates non‐significance, and shaded areas are 95% confidence intervals.

**FIGURE 2:**
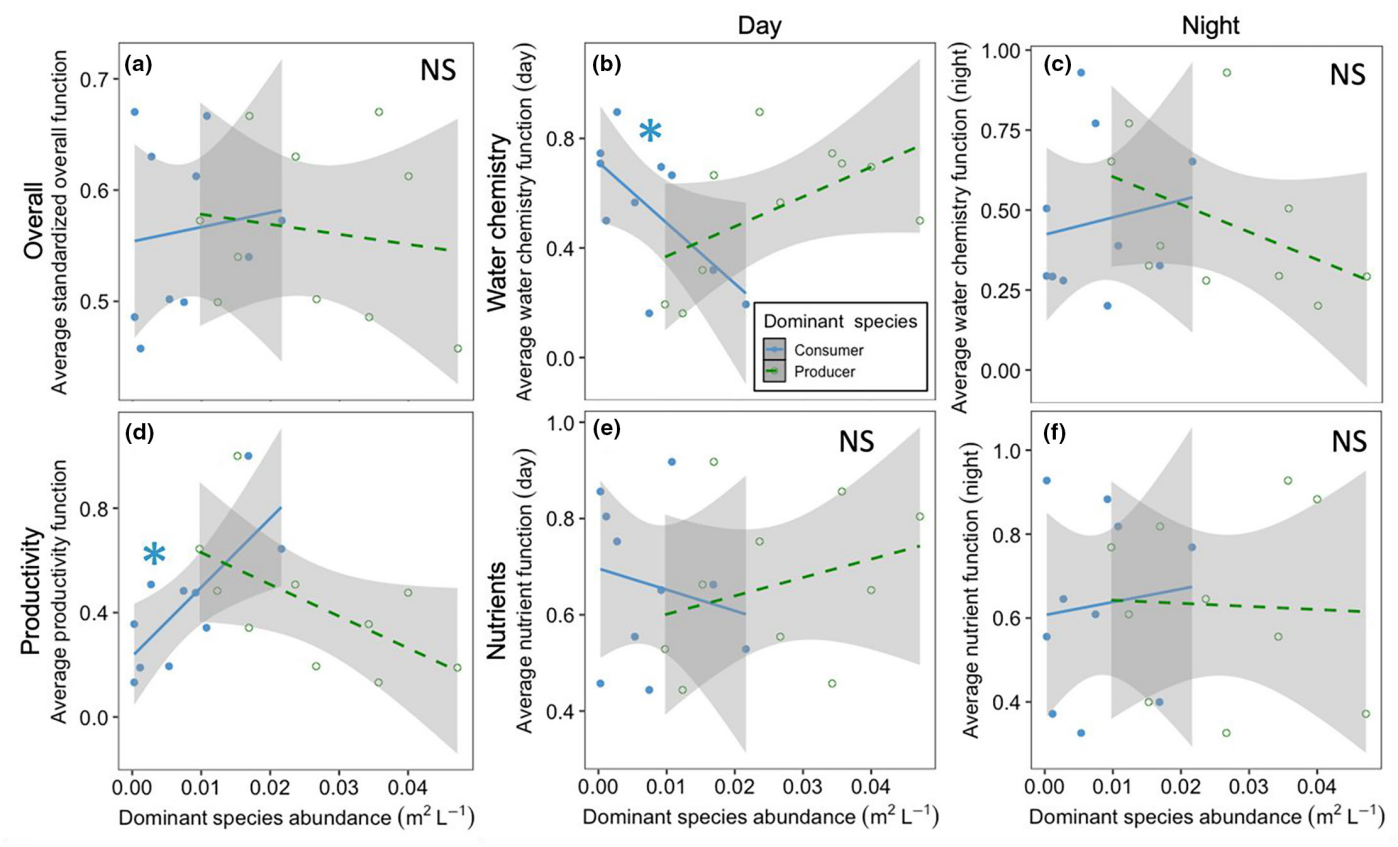
Relationships between the abundances of a dominant producer (green) and a dominant consumer (blue) on averaged rates of (a) overall ecosystem functions as well as groups of functions including change in water chemistry during the (b) day and (c) night, (d) productivity, and change in nutrient levels during the (e) day and (f) night. Abundances of neither the producer *N. oregona* nor the mussel *M. trossulus* were associated with averaged overall ecosystem multifunctionality (the mean of all 12 standardized function values). Dominant consumer abundance, however, showed a positive association with productivity and a negative correlation with daytime water chemistry. The average function of each pool (*N* = 10) is represented in each plot by two points, corresponding to the abundance of the dominant consumer (in blue) and the dominant producer (in green). Asterisks indicate significance, NS indicates non‐significance, and shaded areas are the 95% confidence interval.

**FIGURE 3:**
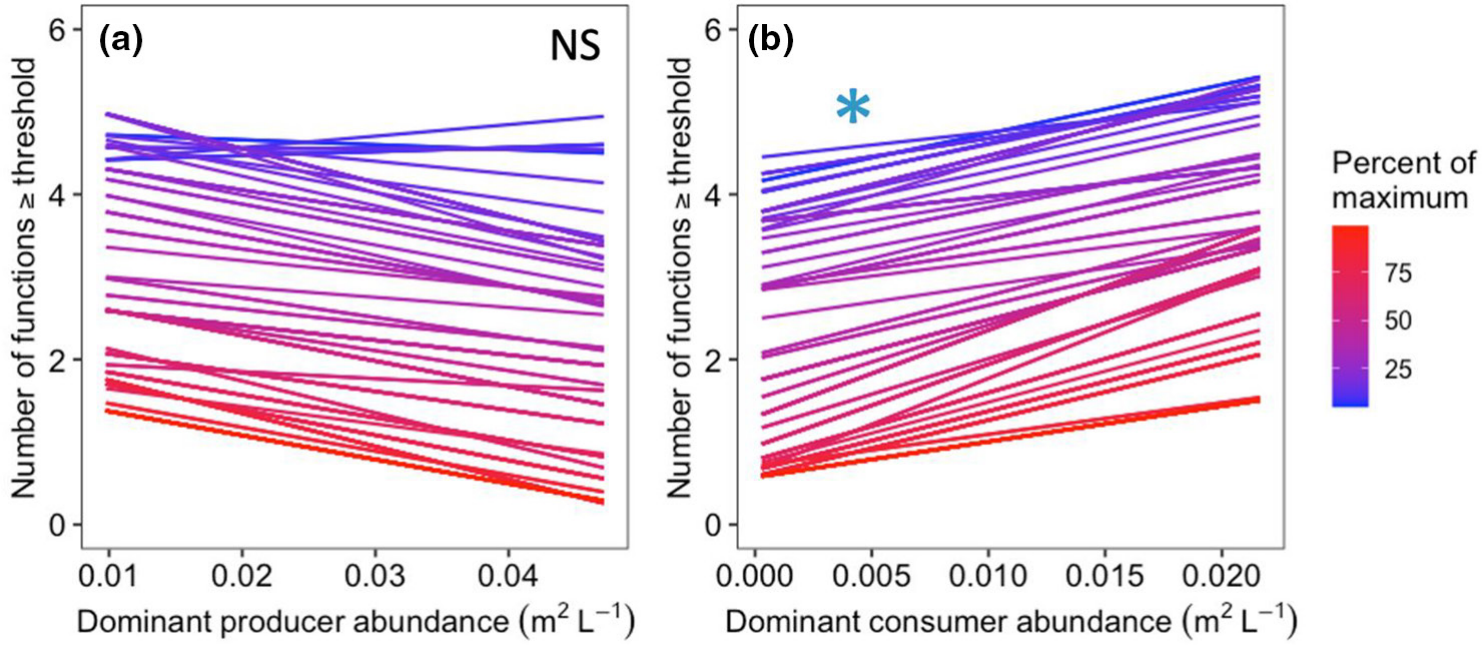
Number of functions exceeded by the (a) dominant producer and (b) dominant consumer based on multiple thresholds to evaluate effects on ecosystem multifunctionality in intact (unmanipulated) tide pools. The abundance of a dominant producer, the alga *Neorhodomela oregona*, was not related to ecosystem multifunctionality, whereas abundance of a dominant consumer, the mussel *Mytilus trossulus*, was positively associated with ecosystem multifunctionality across a wide range of thresholds. Each line indicates the relationship between species abundance and the number of ecosystem functions exceeding a threshold value (indicated by color based on the gradient to the right). Asterisks indicate significance and NS indicates non‐significance.

**FIGURE 4:**
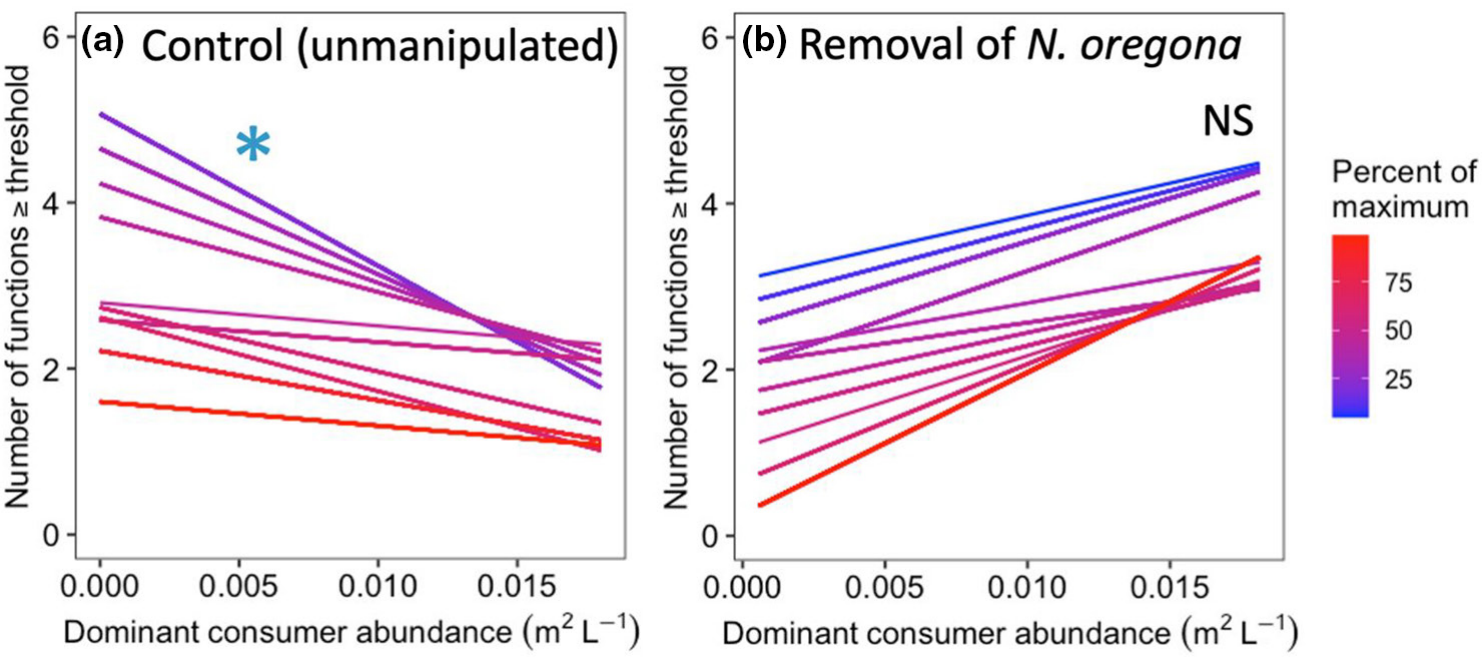
After the removal of the dominant producer, the abundance of the dominant consumer was negatively associated with multifunctionality across a narrow range of thresholds in the (a) control tide pools (with *Neorhodomela oregona* still present) but (b) showed non‐significant positive trends with ecosystem function in pools from which the dominant producer was removed. These analyses follow the multiple threshold approach, as in Figure 3. Asterisks indicate significance and NS indicates non‐significance.

**FIGURE A2:**
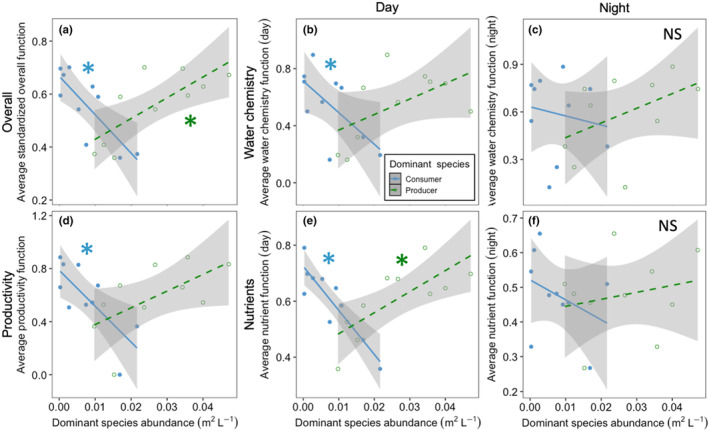
Relationships between the abundances of a dominant consumer (blue) and a dominant producer (green) on averaged rates of (a) overall ecosystem functions, change in water chemistry during the (b) day and (c) night, (d) productivity, and change in nutrient levels during the (e) day and (f) night, using data that have been reflected to establish positive directionality for the relationship between each function and *Neorhodomela oregona* abundance. The abundance of a dominant consumer, the mussel *Mytilus trossulus*, was (a) negatively associated with averaged overall ecosystem multifunctionality, driven by negative relationships with (d) averaged productivity, (b) water chemistry, and (e) nutrient function during the day. The average function of each pool is represented in each plot by a pair of points, corresponding to the abundance of the dominant consumer (in blue) and the dominant producer (in green) in that tide pool. Algal (*N. oregona*) abundance was (a) associated with averaged overall ecosystem multifunctionality (the mean of all 12 standardized function values) in *N* = 10 unmanipulated tide pools, driven most strongly by (e) averaged nutrient function during the day (the mean of the standardized daytime function values of the three nutrients responses). Asterisks indicate significance, NS indicates non‐significance, and shaded areas represent a 95% confidence interval.

**FIGURE A3:**
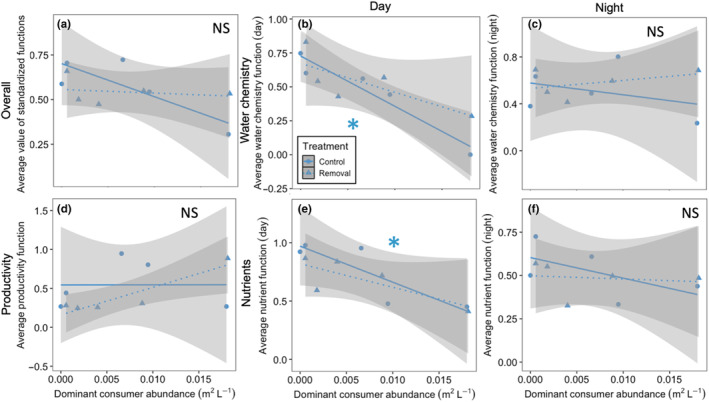
Relationships between the abundances of a dominant consumer on averaged rates of ecosystem functions including (a) overall function, change in water chemistry during the (b) day and (c) night, (d) productivity, and change in nutrient levels during the (e) day and (f) night, separated by treatment group: control (dominant producer present; blue, circles and solid regression lines) and removal (dominant producer removed; blue, triangles and dotted regression lines). Following removal of a dominant alga (*Neorhodomela oregona*), the abundance of mussels (*Mytilus trossulus*) was negatively associated with (b) daytime water chemistry and (e) daytime nutrient function across both treatments. The effect of *M. trossulus* did not differ between tide pools where *N. oregona* was present and pools where *N. oregona* had been removed. Asterisks indicate significance, NS indicates non‐significance, and shaded areas correspond to a 95% confidence interval.

**FIGURE A4:**
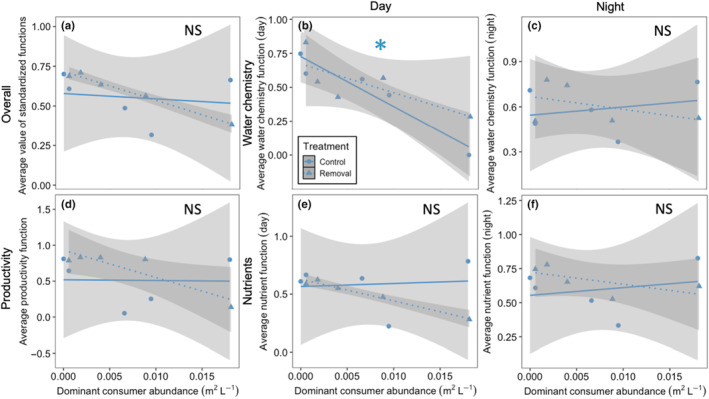
Relationships between the abundances of a dominant consumer in two treatment groups, control (dominant producer present; blue, circles and solid regression lines) and removal (dominant producer removed; blue, triangles and dotted regression lines), on averaged rates of ecosystem functions including (a) overall function, change in water chemistry during the (b) day and (c) night, (d) productivity, and change in nutrient levels during the (e) day and (f) night, using data that have been reflected to ensure positive relationships between each function and dominant producer abundance. Following removal of a dominant alga (*Neorhodomela oregona*), mussel (*Mytilus trossulus*) abundance was (b) associated with daytime water chemistry, but the effect of *M. trossulus* did not differ between tide pools where the dominant producer was present and pools where it had been removed. Asterisks indicate significance, NS indicates non‐significance, and the shaded areas represent a 95% confidence interval.

**FIGURE A5:**
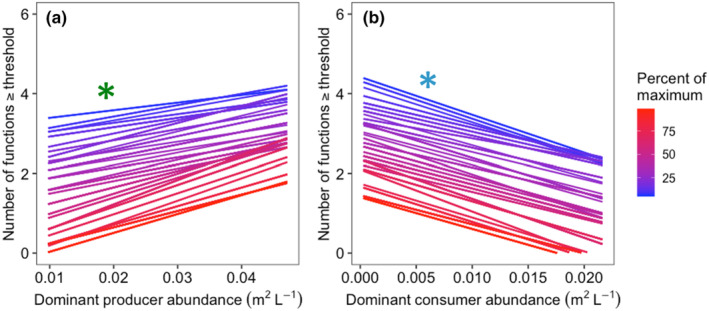
The multiple threshold approach, using data that have been reflected to establish positive directionality between individual functions and *Neorhodomela oregona* abundance, showed the abundance of a dominant producer to be (a) positively associated with ecosystem multifunctionality in tide pools. The abundance of a dominant consumer, the mussel *Mytilus trossulus*, was (b) negatively associated with ecosystem multifunctionality using the same method. Each line indicates the relationship between target species abundance in each tide pool and the number of ecosystem functions in that pool which exceed a certain threshold value, with asterisks included to indicate significance.

**FIGURE A6:**
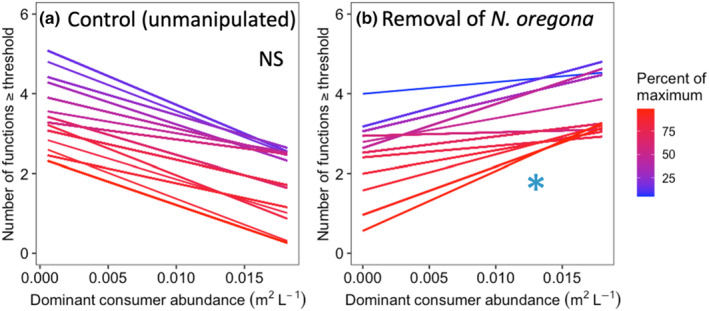
Following the elimination of a dominant alga (*Neorhodomela oregona*) from the removal tide pools, mussel (*Mytilus trossulus*) abundance tended to (a) increase multifunctionality in the control group pools (with *Neorhodomela oregona* still present) but was (b) negatively associated with ecosystem function in the removal group across a small range of thresholds (using reflected data with positive directionality between individual ecosystem functions and *N. oregona* abundance). These analyses follow the multiple threshold approach, where each line indicates the relationship between *Mytilus trossulus* abundance in each tide pool and the number of ecosystem functions in that pool which exceed a certain threshold value, with asterisks indicating significance and NS indicating non‐significance.

All statistical analyses were conducted in R (R‐version 4.0.4; R Core Team, 2013) using linear models (lm), mixed‐effects models (lmer), and the *multifunc* package (Byrnes et al., 2014). We applied the *multifunc* R package (substituting *N. oregona* and *M. trossulus* abundance for species richness) to gauge the effect of individual species rather than overall community diversity (Figures 1, 2, 3, 4, A2, A3, A4, A5, A6). We used liner models to compare the abundance of the dominant consumer to dominant producer abundance. To ensure that any functional effects of the dominant consumer were not confounded by abundance correlations with other producers, we also compared the abundance of the dominant consumer to the total abundance of non‐dominant producers and the abundance of the most widespread non‐dominant producer.

We analyzed the effect of *N. oregona* (the dominant producer) and *M. trossulus* (the dominant consumer) abundance on 12 ecosystem functions in intact, unmanipulated tide pools, as well as the impact of removing *N. oregona* on the functional effect of *M. trossulus*. For each analysis, we began by calculating the effect of the dominant species abundances on each individual functional response in the tide pools (Giling et al., 2019). Next, we standardized the data by dividing each functional response value by the greatest value observed for that function and then calculating the proportion of that maximum value for each functional response (Byrnes et al., 2014; Moi et al., 2021). This standardization method enabled the aggregation of multiple functional responses into values of average functionality (Mouillot et al., 2011) across the suite of ecosystem functions we studied, which we calculated by taking the mean value of all standardized functional values within a single tide pool during a phase of the experiment (pre‐removal or post‐removal). We used the averaging approach on all 12 functions combined as well as subsets of functions, including productivity (net primary productivity and respiration), water chemistry (the rate of pH change and net calcification; both during day and night for four total responses), and nutrient cycling (fluxes of nitrate and nitrite, ammonium, and phosphate; each during day and night for six total metrics).

We also used the standardized data to determine the number of functions in each pool which exceeded the set threshold (Zavaleta et al., 2010), as well as expanded that approach to include all possible thresholds from 5% to 99% (Byrnes et al., 2014). In this multiple threshold approach, the output is the range of potential thresholds for which there is a significant effect of the driver—in this case either dominant producer or dominant consumer abundance—on the number functions exceeding the threshold. A strong dominant species effect is indicated when there is a wide range of thresholds at which its abundance is important in determining the degree of multifunctionality (i.e., the number of functions exceeding a threshold) while a narrow band of significance indicates a weak or negligible effect.

In the analyses on individual functions, averaged functions, and multiple thresholds, we assigned directionality to the response metrics to align with the predicted effects of a dominant producer during the day: higher NCP and respiration were indicated by more positive values, as were higher rates of ecosystem calcification, more positive rates of pH change, and greater nutrient uptake (Table A1). In a second analysis, we repeated the averaging and threshold calculations with all functions denoted as positive (i.e., factors which showed negative trends with dominant producer abundance were “reflected” to become positive; Austin et al., 2021; Figures A2 and A4, A5, A6, Table A1). This was done to remove the possibility that multiple functions would counteract each other based on differing directionality of impact, leading to an underestimate of the effect of the dominant producer on groups of related functions (Giling et al., 2019).

We evaluated the effect of removal of the dominant producer on the functional role of the dominant consumer as follows. Using the lme4 and lmerTest packages (Kuznetsova et al., 2017), we ran mixed‐effects models with each individual ecosystem function as the response and the following fixed effects: dominant consumer abundance (continuous), dominant producer removal treatment (control vs. removal), and time (before vs. after the removal treatment), as well as the consumer abundance:treatment, treatment:time, and consumer abundance:treatment:time interactions; tide pool was included as a random effect. The three‐way interaction (mussel abundance:treatment:time) is of particular interest, as it represents the potential shift in dominant consumer function when the dominant producer is present versus absent. The two‐way interaction between consumer abundance and time was not significant across functions and was therefore removed from the analysis. Data were log‐ or inverse‐transformed where necessary (daytime ammonium and phosphate data, respectively) to satisfy the normality assumptions of mixed models.

## 3 RESULTS

### 3.1 Opposing functional effects of the dominant producer and dominant consumer

We found that increases in both dominant producer and dominant consumer abundance were associated with changes in individual ecosystem functions in almost uniformly opposite directions (Figure 1). Increases in dominant producer abundance were only associated with changes in two of the 12 ecosystem functions, reducing the respiration rate (*F*_1,8_ = 9.34, *p* = .016) and increasing the rate of daytime net ecosystem calcification (*F*_1,8_ = 10.01, *p* = .013). Increases in dominant consumer abundance were associated with changes in three of the 12 ecosystem functions studied, including increases in net community productivity (*F*_1,8_ = 5.63, *p* = .045) and respiration (*F*_1,8_ = 6.49, *p* = .034), as well as a reduction in the rate of daytime net ecosystem calcification (*F*_1,8_ = 7.01, *p* = .029), while all other functions were not significantly related to dominant consumer abundance (Table 1). Virtually all (11/12) of the relationships between functions and dominant producer abundance were in the opposite direction from the trends of the relationships between those same functions and dominant consumer abundance, though the majority of the relationships between functions and the abundances of the two species were not significant. The only function that did not switch directionality from one species to the other was the rate of change in phosphate concentrations during the day, but it was not significantly related to the abundance of either species (*p* > .5 for *N. oregona* and *p* > .9 for *M. trossulus*). The slopes of the relationships between dominant producer abundance and individual ecosystem functions were negative (i.e., increases in abundance were associated with declines in functioning) for seven functions and positive for five functions (including all significant effects and non‐significant trends), whereas the directionality of relationships between ecosystem function and dominant consumer abundance was generally positive (eight positive vs. four negative; similarly including both significant and non‐significant trends).

**TABLE 1:** Relationships between the abundances of the dominant producer (the alga *Neorhodomela oregona*) and consumer (the mussel *Mytilus trossulus*) and 12 individual ecosystem functions: net community production; daytime net ecosystem calcification and pH change; community respiration; nighttime net ecosystem calcification and pH change; daytime ammonium accumulation, nitrate + nitrite uptake, and phosphate uptake; and nighttime ammonium accumulation, nitrate + nitrite uptake, and phosphate uptake.

Function	Function category	Factor	Sum of squares	dF	*F* value	*p* Value
NCP	Productivity	*N. oregona* abundance (m^2^ L^−1^)	3.3516	1,8	1.0171	.3427
		*M. trossulus* abundance (m^2^ L^−1^)	12.275	1,8	5.6315	**.04503**
Respiration		*N. oregona* abundance (m^2^ L^−1^)	13.955	1,8	9.3433	**.01566**
		*M. trossulus* abundance (m^2^ L^−1^)	11.605	1,8	6.4932	**.03427**
Rate of pH change (day)	Water chemistry (day)	*N. oregona* abundance (m^2^ L^−1^)	0.000607	1,8	0.0557	.8193
		*M. trossulus* abundance (m^2^ L^−1^)	0.007396	1,8	0.7363	.4158
NEC (day)		*N. oregona* abundance (m^2^ L^−1^)	6.2251	1,8	10.011	**.01331**
		*M. trossulus* abundance (m^2^ L^−1^)	5.2317	1,8	7.013	**.02934**
Rate of pH change (night)	Water chemistry (night)	*N. oregona* abundance (m^2^ L^−1^)	0.0005667	1,8	1.0009	.3464
		*M. trossulus* abundance (m^2^ L^−1^)	0.0000757	1,8	0.1205	.7374
NEC (night)		*N. oregona* abundance (m^2^ L^−1^)	0.27432	1,8	2.0691	.1883
		*M. trossulus* abundance (m^2^ L^−1^)	0.03274	1,8	0.2012	.6657
Rate of ammonium concentration change (day)	Nutrients (day)	*N. oregona* abundance (m^2^ L^−1^)	8.0966	1,8	3.2782	.1078
		*M. trossulus* abundance (m^2^ L^−1^)	16.39	1,8	0.3751	.5572
Rate of nitrate + nitrite concentration change (day)		*N. oregona* abundance (m^2^ L^−1^)	0.2015	1,8	0.483	.5068
		*M. trossulus* abundance (m^2^ L^−1^)	0.61446	1,8	1.6804	.231
Rate of phosphate concentration change (day)		*N. oregona* abundance (m^2^ L^−1^)	0.2253	1,8	0.451	.5208
		*M. trossulus* abundance (m^2^ L^−1^)	0.0044	1,8	0.0084	.9292
Rate of ammonium concentration change (night)	Nutrients (night)	*N. oregona* abundance (m^2^ L^−1^)	0.773	1,8	0.0714	.796
		*M. trossulus* abundance (m^2^ L^−1^)	6.252	1,8	0.6169	.4548
Rate of nitrate + nitrite concentration change (night)		*N. oregona* abundance (m^2^ L^−1^)	0.01313	1,8	0.0593	.8137
		*M. trossulus* abundance (m^2^ L^−1^)	0.00712	1,8	0.032	.8624
Rate of phosphate concentration change (night)		*N. oregona* abundance (m^2^ L^−1^)	0.0465	1,8	0.0409	.8447
		*M. trossulus* abundance (m^2^ L^−1^)	0.0645	1,8	0.0569	.8174

*Note*: Significant relationships related to either dominant species are presented in bold and all other effects were not significant.

Abundances of neither the dominant producer nor the dominant consumer were associated with average ecosystem multifunctionality (*F*_1,8_ = 0.18, *p* = .686, and *F*_1,8_ = 0.12, *p* = .741, respectively), though certain groups of functions were affected in opposing directions by the different species (Figure 2). We observed a negative, though only marginally significant, trend in the relationship between dominant producer abundance and productivity (*F*_1,8_ = 4.59, *p* = .065), while consumer abundance and productivity were positively associated (*F*_1,8_ = 9.92, *p* = .013). Dominant producer abundance displayed a positive non‐significant trend in its relationship with water chemistry during the day (*F*_1,8_ = 3.64, *p* = .093; Table A2), compared to a negative relationship between dominant consumer abundance and daytime changes in water chemistry (*F*_1,8_ = 5.79, *p* = .043; Table A3).

We found that ecosystem multifunctionality was associated with dominant consumer abundance, but not dominant producer abundance, in unmanipulated tide pools using the multiple threshold approach (Figure 3). The abundance of the dominant consumer was positively associated with ecosystem function by the multiple threshold approach over two distinct ranges of thresholds (threshold values 51%–56%, 64%–77%; *p* < .05). In those same tide pools, the dominant producer was not associated with ecosystem multifunctionality (*p* > .1), though the relationship between producer abundance and multifunctionality tended to be negative across thresholds. Results for identical analyses using the reflected data are shown in Figures A2, A4, and A5.

### 3.2 Impact of dominant producer removal on the functional effect of the dominant consumer

Following the removal of the dominant producer, the relationships between dominant consumer abundance and several individual functions, particularly nutrient fluxes, differed markedly between tide pools where the producer had been removed and pools with the producer still present. The associations between dominant consumer abundance and daytime fluxes of ammonium and nitrate + nitrite (*F*_2,6_ = 25.15, *p* = .001, and *F*_2,6_ = 5.36, *p* = .049, respectively; dominant consumer abundance:treatment:time) differed between pools where the dominant producer had been removed and control pools where it was still present. Changes in ammonium fluxes were also associated with the removal of the dominant producer, irrespective of dominant consumer abundance, (*F*_1,5_ = 7.10, *p* = .041; treatment:time). In addition, both ammonium (*F*_1,10_ = 17.82, *p* = .002) and nitrate + nitrite (*F*_1,12_ = 8.09, *p* = .015; dominant consumer abundance:treatment) fluxes were associated with an interaction between dominant consumer abundance and treatment group. Dominant consumer abundance was associated with increased NCP (*F*_1,12_ = 6.92, *p* = .022), as well as more rapid acidification (i.e., negative pH change) and greater ammonium accumulation during the day (*F*_1,9_ = 8.16, *p* = .02; *F*_1,10_ = 38.30, *p* < .001), regardless of time or removal treatment.

The dominant consumer tended to reduce overall averaged ecosystem function after dominant producer removal (*F*_1,6_ = 4.88, *p* = .069; Figure A3), though the effect was not significant, driven by negative associations between consumer abundance and daytime water chemistry (*F*_1,6_ = 23.06, *p* = .003) and nutrient fluxes (*F*_1,6_ = 12.25, *p* = .012). However, we did not find evidence of an interaction between the removal of the dominant producer and the effect of dominant consumer abundance on averaged ecosystem function or any individual set of functions (*p* > .1; dominant consumer abundance:treatment; Table A4).

The relationship between dominant consumer abundance and ecosystem multifunctionality, as assessed using the multiple threshold approach, differed depending on whether the dominant producer was present (Figure 4). In the experimental tide pools, dominant consumer abundance was negatively related to ecosystem multifunctionality over a narrow band of thresholds where the dominant producer was present (threshold values 5%–23%; *p* < .05), while the relationships between consumer abundance and multifunctionality tended to be positive in the pools where the producer had been removed (NS; *p* > .2). Results for analyses on the reflected data are shown in Figure A6.

## 4 DISCUSSION

We found that the relationships between the abundances of each dominant species and individual ecosystem functions, as well as groups of functions, were consistently in opposing directions. This pattern may reflect the differing roles of producers and consumers in supporting overall ecosystem function, in which different trophic levels tend to contribute to certain functions, or types of functions, in specific ways (e.g., producers raising pH during the day or absorbing nutrients; Aquilino et al., 2009; Bracken et al., 2018). However, dominant consumer abundance was related to many of the functions in the direction predicted to be associated with a producer. This producer‐like effect of the dominant consumer may reflect an indirect effect in which the consumer is affecting ecosystem function through facilitation of non‐dominant producers (Aquilino et al., 2009), the total abundance of which was found to be positively related to dominant consumer abundance (*F*_1,8_ = 6.12, *p* = .038). This potential indirect effect on ecosystem function by a sessile, filter‐feeding consumer may differ from that of mobile, herbivorous consumers, which may more strongly impact producers via herbivory, or conversely, herbivores may preferentially consume the dominant producer and enable other producers to flourish (Altieri et al., 2009). The opposing effects of *N. oregona* and *M. trossulus* may be more specifically indicative of the well‐documented interactions between tide pool algae and mussels, particularly in terms of nutrient cycling (Bracken & Nielsen, 2004; Pfister, 2007). Either way, the nearly uniform counter‐directionality of effects between these two dominant species suggests an ecological equilibrium, maintained by the presence of both species, which may be disrupted if one species is lost.

Interestingly, we found that there was a directional change in the relationship between dominant consumer abundance and ecosystem multifunctionality, from positive during the pre‐removal sampling to negative in the control pools in the post‐removal sampling (i.e., with the dominant producer still present; Figure A1). This directional change might have been driven by shifts in temperature and light levels between samplings related to changes in weather: mean temperature and light measurements of 20.7°C and 524 μmol m^−2^ s^−1^ prior to removal dropped to 15.3°C and 64 μmol m^−2^ s^−1^ during the post‐removal sampling in all tide pools studied across both treatment groups (S.A. Mahanes, M.E.S. Bracken, C.J.B. Sorte, unpublished data). This decline in temperature could have altered the functional effect of the dominant consumer by affecting metabolic rate (Bracken et al., 2022; Tagliarolo et al., 2012). Additionally, if the dominant consumer is indirectly affecting NCP and ecosystem function more broadly by facilitating non‐dominant producers, shifts in light availability may disrupt those indirect effects (Aquilino et al., 2009). The shift in effect direction highlights the potential for changes in the functional impacts of individual species under different environmental contexts and raises intriguing questions about how the ecological roles of abundant species may shift across timescales, driven by changes in weather patterns, seasonal cycles, or long‐term environmental change.

We found that the direction of the effect of dominant consumer abundances on ecosystem multifunctionality differed between treatment groups, suggesting that the presence of the dominant producer affected the functional effect of the dominant consumer. The tide pools where the dominant producer had been removed tended to have more positive rates of pH change relative to pools with the dominant producer still present, suggesting that either (1) *N. oregona* is largely functioning as a consumer in low light conditions, reducing pH in the pools and restricting calcification, or that (2) non‐dominant producers were released from photosynthetic limitation by the removal of the abundant alga. The removal treatment pools tended to have positive relationships between dominant consumer abundance and calcification, which may be related to increased pH in those pools relative to the control group. We found no difference in the effect of consumer abundance and nutrient fluxes in the producer removal pools and the control pools, but the potential disruption of reciprocal nutrient cycling between a dominant consumer and a dominant producer presents an intriguing mechanism for an interactive impact of dominant producer loss.

We did not find a comparable effect using the averaging method on un‐reflected data, either on groups of functions or overall, which may be due to methodological differences between the averaging and multiple threshold approaches: the multiple threshold method is weighted toward consistent baseline levels across functions, rather than exceptionally high levels of individual functions which may elevate the overall average (Manning et al., 2018). The conclusions drawn from the results of either approach may be limited in their scope due to the relatively small sample size of the experiment. Our reasoning for grouping certain functions together is that related functions may be similarly associated with species abundances. Studies have shown, for example, that calcification rates tend to be higher in relatively high‐pH conditions (Semesi et al., 2009; Wahl et al., 2018). However, there may be intergroup interactions occurring among ecosystem functions as well: respiration can directly affect pH by modifying CO_2_ levels (Krause‐Jensen et al., 2015), and productivity and respiration may be intertwined with nutrient cycling due to potential oxygen limitation of nitrification (Joo et al., 2005; Pfister & Altabet, 2019). We focused on the un‐reflected data but included identical analyses on the reflected data in the supplement for additional context (Figures A2, A4, A5, A6). The rationale for reflecting the data, where necessary, to produce a positive slope with dominant producer abundance in unmanipulated tide pools was to ensure that significant effects, overall or in groups of functions, were not being obscured by opposing effects. We found this to be the case with dominant producer abundance and daytime nutrient fluxes in intact tide pools: both ammonium and phosphate accumulation tended to be more positive in pools with greater dominant producer abundance, while nitrate and nitrite tended to accumulate more slowly in those pools, resulting in an association between dominant producer abundance and daytime nutrient function in unmanipulated tide pools with the reflected data but no corresponding effect in the un‐reflected data.

We used approaches designed for evaluating diversity–multifunctionality relationships to focus on the effects of dominant species on multifunctionality in tide pools, but the methods employed in this study could be applicable across a wide range of ecosystems. For example, the patterns we uncovered regarding the opposing effects of species from different trophic levels and potential interactive functional impacts of dominant species could be evaluated in other ecosystems with both dominant producers and consumers present (e.g., forests with a highly abundant variety of tree and a dominant fungal species) to determine whether those trends are widespread or unique to marine ecosystems where consumers are often dominant. This study focused explicitly on dominant species, but less abundant species can also play considerable roles in structuring the community and driving ecological function. Mariotte (2014) highlights the ecological importance of non‐dominant species and other recent studies have shown their ability to reduce the effect of drought on soil communities (Mariotte et al., 2015), stabilize food webs (Shao et al., 2016), and impact community composition (Bracken & Low, 2012). Considerable effort has been devoted to identifying species which drive critical functions in ecosystems, including keystone species (Paine, 1966), foundation species (Ellison, 2019; Fields & Silbiger, 2022), and ecosystem engineers (Losapio et al., 2021). Dominant species may have similarly substantial impacts on the ecosystem by virtue of their abundance (Grime, 1998; Orwin et al., 2014), and more research comparing the impacts of dominant species loss to the loss of species of other functional types (e.g., foundation, species, keystone species, ecosystem engineers, or non‐dominant species) may further illuminate the ecological role of dominant species. Additionally, focusing on the impacts of individual species may inform biodiversity–multifunctionality research, in which the relative importance of sampling effects (i.e., a greater pool of species increases the likelihood that an impactful species will be present to drive ecosystem function) and complementarity (i.e., the differences in functional traits among species, rather than the traits of a single species, strongly impacts ecosystem function) in driving the species diversity–ecosystem multifunctionality relationship is a constant question. Such research into the potential for differential ecological impacts of the loss of species of different functional types is pertinent and timely in the context of widespread biodiversity loss, and may be instrumental in understanding how biodiversity loss will manifest across ecosystems. The approach applied here could advance our understanding of the roles of individual species—and their interactions—in mediating multiple ecosystem functions. Understanding both the role of abundant species in ecosystems and their susceptibility to global change will be critical to forecasting future alterations in the functioning of these ecosystems.

## AUTHOR CONTRIBUTIONS

**Samuel A. Mahanes:** Conceptualization (equal); formal analysis (lead); methodology (equal); writing – original draft (lead). **Cascade J. B. Sorte:** Conceptualization (equal); methodology (equal); writing – original draft (supporting). **Matthew E. S. Bracken:** Conceptualization (equal); methodology (equal); writing – original draft (supporting).

## CONFLICT OF INTEREST STATEMENT

The authors declare that there are no competing interests.

## Supporting information


Appendix S1

## Data Availability

The datasets used in this study are available on the Dryad Digital Repository: doi:10.7280/D1J971.

## Appendix

**TABLE A1:** We assigned directionality to each of the 12 ecological functions based on the predicted effects of a dominant producer during the day.

Functions	Function group	Units	Positive direction assigned (un‐reflected)	Rationale	Positive direction assigned (reflected)
Net community productivity (NCP)	Productivity	mg O_2_ L^−1^ h^−1^	Increase in O_2_	Dominant producers are expected to increase net primary productivity during the day	Decrease in O_2_
Respiration	Productivity	mg O_2_ L^−1^ h^−1^	Decrease in O_2_	Dominant producers are expected to increase resipration during the day	Increase in O_2_
Rate of pH change (day)	Water chemistry (day)	units h^−1^	Increase in pH	Dominant producers are expected to raise pH through photosynthesis by extracting inorganic carbon from the water column during the day	Increase in pH
Rate of pH change (night)	Water chemistry (night)	units h^−1^	Increase in pH		Decrease in pH
Net ecosystem calcification (NEC, day)	Water chemistry (day)	mmol CaCO_3_ m^−2^ h^−1^	Positive NEC	Dominant producers are expected to increase NEC during the day by producing a higher pH environment that is more suitable to calcification	Positive NEC
Net ecosystem calcification (NEC, night)	Water chemistry (night)	mmol CaCO_3_ m^−2^ h^−1^	Positive NEC		Negative NEC
Ammonium flux (day)	Nutrients (day)	μmol ${\mathrm{NH}}_{4}^{+}$ L^−1^ h^−1^	Decrease in concentration	Dominant producers are expected to take up nutrients during the day, leading to reduced nutrient concentration in the water column	Decrease in concentration
Ammonium flux (night)	Nutrients (night)	μmol ${\mathrm{NH}}_{4}^{+}$ L^−1^ h^−1^	Decrease in concentration		Increase in concentration
Nitrate + nitrite flux (day)	Nutrients (day)	μmol (${\mathrm{NO}}_{3}^{-}$ + ${\mathrm{NO}}_{2}^{-}$) L^−1^ h^−1^	Decrease in concentration		Increase in concentration
Nitrate + nitrite flux (night)	Nutrients (night)	μmol (${\mathrm{NO}}_{3}^{-}$ + ${\mathrm{NO}}_{2}^{-}$) L^−1^ h^−1^	Decrease in concentration		Increase in concentration
Phosphate flux (day)	Nutrients (day)	μmol ${\mathrm{PO}}_{4}^{3-}$ L^−1^ h^−1^	Decrease in concentration		Decrease in concentration
Phosphate flux (night)	Nutrients (night)	μmol ${\mathrm{PO}}_{4}^{3-}$ L^−1^ h^−1^	Decrease in concentration		Decrease in concentration

*Note*: We also reflected the data based on the associations between dominant producer abundance and each individual function in intact tide pools to avoid positive and negative values obscuring overall patterns (Figures A2 and A4, A5, A6).

**TABLE A2:** Analysis of the associations between the abundance of a dominant alga (*Neorhodomela oregona*) and averaged sets of ecosystem functions (all 12 functions, followed by subsets of related functions) in *N* = 10 unmanipulated tide pools near Sitka, AK.

Function set	Factor	Sum of squares	df	*F* value	*p* Value
Overall function	*N. oregona* abundance (m^2^ L^−1^)	0.00118	1,8	0.1764	.6856
Productivity	*N. oregona* abundance (m^2^ L^−1^)	0.21754	1,8	4.5886	.0646
Water chemistry (day)	*N. oregona* abundance (m^2^ L^−1^)	0.17233	1,8	3.6367	.0929
Water chemistry (night)	*N. oregona* abundance (m^2^ L^−1^)	0.10967	1,8	2.0667	.1885
Nutrients (day)	*N. oregona* abundance (m^2^ L^−1^)	0.021259	1,8	0.7426	.4139
Nutrients (night)	*N. oregona* abundance (m^2^ L^−1^)	0.0008	1,8	0.015	.9055

**TABLE A3:** Analysis of the associations between the abundance of a dominant consumer (*Mytilus trossulus*) and averaged sets of ecosystem functions (all 12 functions, followed by subsets of related functions) in tide pools.

Function set	Factor	Sum of squares	df	*F* value	*p* Value
Overall function	*M. trossulus* abundance (m^2^ L^−1^)	0.000791	1,8	0.1174	.7407
Productivity	*M. trossulus* abundance (m^2^ L^−1^)	0.33013	1,8	9.9034	**.0137**
Water chemistry (day)	*M. trossulus* abundance (m^2^ L^−1^)	0.23165	1,8	5.7951	**.0427**
Water chemistry (night)	*M. trossulus* abundance (m^2^ L^−1^)	0.01374	1,8	0.2112	.6581
Nutrients (day)	*M. trossulus* abundance (m^2^ L^−1^)	0.009095	1,8	0.3017	.5978
Nutrients (night)	*M. trossulus* abundance (m^2^ L^−1^)	0.00457	1,8	0.0867	.7759

*Note*: The significant values have been bolded.

**TABLE A4:** Analyses comparing the abundance of a dominant consumer (*Mytilus trossulus*) to averaged sets of ecosystem functions (all 12 functions, followed by subsets of related functions) in tide pools (*N* = 5 controls with *Neorhodomela oregona* present and *N* = 5 with the dominant alga removed).

Function set	Factor	Sum of squares	df	*F* value	*p* Value
Overall function	*M. trossulus* abundance	0.046759	1,6	4.8849	.06913
	Treatment	0.002673	1,6	0.2793	.61615
	*M. trossulus* abundance*Treatment	0.028573	1,6	2.985	.13478
Productivity	*M. trossulus* abundance	0.1249	1,6	1.7003	.24
	Treatment	0.05275	1,6	0.7181	.4293
	*M. trossulus* abundance*Treatment	0.13089	1,6	1.7818	.2303
Water chemistry (day)	*M. trossulus* abundance	0.36551	1,6	23.057	**.002995**
	Treatment	0.00692	1,6	0.4366	.533312
	*M. trossulus* abundance*Treatment	0.02598	1,6	1.6387	.247767
Water chemistry (night)	*M. trossulus* abundance	0.001436	1,6	0.0392	.8496
	Treatment	0.011795	1,6	0.3221	.5909
	*M. trossulus* abundance*Treatment	0.029854	1,6	0.8152	.4014
Nutrients (day)	*M. trossulus* abundance	0.27876	1,6	12.249	**.01283**
	Treatment	0.01515	1,6	0.6657	.44573
	*M. trossulus* abundance*Treatment	0.012084	1,6	0.531	.49364
Nutrients (night)	*M. trossulus* abundance	0.020816	1,6	1.2836	.3005
	Treatment	0.00342	1,6	0.2109	.6622
	*M. trossulus* abundance*Treatment	0.010482	1,6	0.6464	.4521

*Note*: Dominant consumer abundance was negatively associated with daytime water chemistry and daytime nutrient function across both treatments and the effect did not differ between treatment groups. The significant values have been bolded.

## References

[ece310342-bib-0001] Aarssen, L. W. (1997). High productivity in grassland ecosystems: Effected by species diversity or productive species? Oikos, 80(1), 183–184.

[ece310342-bib-0002] Aiuppa, A. , Hall‐Spencer, J. M. , Milazzo, M. , Turco, G. , Caliro, S. , & di Napoli, R. (2021). Volcanic CO_2_ seep geochemistry and use in understanding ocean acidification. Biogeochemistry, 152(1), 93–115. 10.1007/A10533-020-00737-9

[ece310342-bib-0003] Altieri, A. H. , Silliman, B. R. , & Bertness, M. D. (2007). Hierarchical organization via a facilitation cascade in intertidal cordgrass bed communities. American Naturalist, 169(2), 195–206. 10.1086/510603 17211804

[ece310342-bib-0004] Altieri, A. H. , Trussell, G. C. , Ewanchuk, P. J. , Bernatchez, G. , & Bracken, M. E. S. (2009). Consumers control diversity and functioning of a natural marine ecosystem. PLoS One, 4(4), e5291. 10.1371/journal.pone.0005291 19384410 PMC2668074

[ece310342-bib-0005] Andersson, A. J. , & Gledhill, D. (2013). Ocean acidification and coral reefs: Effects on breakdown, dissolution, and net ecosystem calcification. Annual Review of Marine Science, 5, 321–348. 10.1146/annurev-marine-121211-172241 22881351

[ece310342-bib-0006] Angelini, C. , Altieri, A. H. , Silliman, B. R. , & Bertness, M. D. (2011). Interactions among foundation species and their consequences for community organization, biodiversity, and conservation. Bioscience, 61(10), 782–789. 10.1525/bio.2011.61.10.8

[ece310342-bib-0007] Angelini, C. , & Silliman, B. R. (2014). Secondary foundation species as drivers of trophic and functional diversity: Evidence from a tree‐epiphyte system. Ecology, 95(1), 185–196. 24649658 10.1890/13-0496.1

[ece310342-bib-0008] Aquilino, K. M. , Bracken, M. E. S. , Faubel, M. N. , & Stachowicz, J. J. (2009). Local‐scale nutrient regeneration facilitates seaweed growth on wave‐exposed rocky shores in an upwelling system. Limnology and Oceanography, 54(1), 309–317.

[ece310342-bib-0009] Asmus, H. , Asmus, R. M. , & Zubillaga, G. F. (1995). Do mussel beds intensify the phosphorus exchange between sediment and tidal waters? Ophelia, 41(1), 37–55. 10.1080/00785236.1995.10422036

[ece310342-bib-0010] Austin, A. N. , Hansen, J. P. , Donadi, S. , Bergströ, U. , Eriksson, B. K. , Sundblad, G. , & Eklö, J. S. (2021). Synergistic effects of rooted aquatic vegetation and drift wrack on ecosystem multifunctionality. Ecosystems, 24, 1670–1686. 10.1007/A10021-021-0060

[ece310342-bib-0011] Bellard, C. , Bertelsmeier, C. , Leadley, P. , Thuiller, W. , & Courchamp, F. (2012). Impacts of climate change on the future of biodiversity. Ecology Letters, 15(4), 365–377. 10.1111/j.1461-0248.2011.01736.x 22257223 PMC3880584

[ece310342-bib-0012] Braby, C. E. , & Somero, G. N. (2006). Ecological gradients and relative abundance of native (*Mytilus trossulus*) and invasive (*Mytilus galloprovincialis*) blue mussels in the California hybrid zone. Marine Biology, 148(6), 1249–1262. 10.1007/s00227-005-0177-0

[ece310342-bib-0013] Bracken, M. E. S. (2004). Invertebrate‐mediated nutrient loading increases growth of an intertidal macroalga. Journal of Phycology, 40(6), 1032–1041. 10.1111/j.1529-8817.2004.03106.x

[ece310342-bib-0014] Bracken, M. E. S. , & Low, N. H. N. (2012). Realistic losses of rare species disproportionately impact higher trophic levels. Ecology Letters, 15(5), 461–467. 10.1111/j.1461-0248.2012.01758.x 22381064

[ece310342-bib-0015] Bracken, M. E. S. , Miller, L. P. , Mastroni, S. E. , Lira, S. M. , & Sorte, C. J. B. (2022). Accounting for variation in temperature and oxygen availability when quantifying marine ecosystem metabolism. Scientific Reports, 12(1), 825. 10.1038/A41598-021-04685-8 35039551 PMC8763951

[ece310342-bib-0016] Bracken, M. E. S. , & Nielsen, K. J. (2004). Diversity of intertidal macroalgae increases with nitrogen loading by invertebrates. Ecology, 85(10), 2828–2836.

[ece310342-bib-0017] Bracken, M. E. S. , Silbiger, N. J. , Bernatchez, G. , & Sorte, C. J. B. (2018). Primary producers may ameliorate impacts of daytime CO_2_ addition in a coastal marine ecosystem. PeerJ, 2018(5), e4739. 10.7717/peerj.4739 PMC5949060 29761055

[ece310342-bib-0018] Bracken, M. E. S. , & Williams, S. L. (2013). Realistic changes in seaweed biodiversity affect multiple ecosystem functions on a rocky shore. Ecology, 94(9), 1944–1954. 24279266 10.1890/12-2182.1

[ece310342-bib-0019] Brun, P. , Violle, C. , Mouillot, D. , Mouquet, N. , Enquist, B. J. , Munoz, F. , Münkemüller, T. , Ostling, A. , Zimmermann, N. E. , & Thuiller, W. (2022). Plant community impact on productivity: Trait diversity or key(stone) species effects? Ecology Letters, 25(4), 913–925. 10.1111/ele.13968 35064626 PMC9305544

[ece310342-bib-0020] Bruno, J. F. , Lee, S. C. , Kertesz, J. S. , Carpenter, R. C. , Long, Z. T. , Emmett Duffy Bruno, J. , Bruno, J. F. , & Lee, S. C. (2006). Partitioning the effects of algal species identity and richness on benthic marine primary production. Oikos, 115, 170–178.

[ece310342-bib-0021] Byrnes, J. E. K. , Gamfeldt, L. , Isbell, F. , Lefcheck, J. S. , Griffin, J. N. , Hector, A. , Cardinale, B. J. , Hooper, D. U. , Dee, L. E. , & Emmett Duffy, J. (2014). Investigating the relationship between biodiversity and ecosystem multifunctionality: Challenges and solutions. Methods in Ecology and Evolution, 5(2), 111–124. 10.1111/2041-210X.12143

[ece310342-bib-0022] Camp, E. F. , Suggett, D. J. , Gendron, G. , Jompa, J. , Manfrino, C. , & Smith, D. J. (2016). Mangrove and seagrass beds provide different biogeochemical services for corals threatened by climate change. Frontiers in Marine Science, 3, 1–16. 10.3389/fmars.2016.00052

[ece310342-bib-0023] Cardinale, B. J. , Srivastava, D. S. , Duffy, J. E. , Wright, J. P. , Downing, A. L. , Sankaran, M. , & Jouseau, C. (2006). Effects of biodiversity on the functioning of trophic groups and ecosystems. Nature, 443, 989–992. 17066035 10.1038/nature05202

[ece310342-bib-0024] Cardinale, B. J. , Wright, J. P. , Cadotte, M. W. , Carroll, I. T. , Hector, A. , Srivastava, D. S. , Loreau, M. , & Weis, J. J. (2007). Impacts of plant diversity on biomass production increase through time because of species complementarity. Proceedings of the National Academy of Sciences of the United States of America, 104, 18123–18128. 17991772 10.1073/pnas.0709069104 PMC2084307

[ece310342-bib-0025] Cowling, R. M. (1983). The occurrence of C3 and C4 grasses in fynbos and allied shrublands in the South Eastern Cape. Oecologia, 58, 121–127. 28310656 10.1007/BF00384551

[ece310342-bib-0026] Dickson, A. G. , Sabine, C. L. , & Christian, J. R. (2007). *Guide to best practices for ocean CO* _ *2* _ *measurements*. PICES Special Publication, 3.

[ece310342-bib-0027] Ellison, A. M. (2019). Foundation species, non‐trophic interactions, and the value of being common. IScience, 13, 254–268. 10.1016/j.isci 30870783 PMC6416672

[ece310342-bib-0028] Ellison, A. M. , Buckley, H. L. , Case, B. S. , Cardenas, D. , Duque, Á. J. , Lutz, J. A. , Myers, J. A. , Orwig, D. A. , & Zimmerman, J. K. (2019). Species diversity associated with foundation species in temperate and tropical forests. Forests, 10(2), 128. 10.3390/f10020128

[ece310342-bib-0029] Fields, J. B. , & Silbiger, N. J. (2022). Foundation species loss alters multiple ecosystem functions within temperate tidepool communities. Marine Ecology Progress Series, 683, 1–19. 10.3354/mepA13978

[ece310342-bib-0030] Gamfeldt, L. , Hillebrand, H. , & Jonsson, P. R. (2008). Multiple functions increase the importance of biodiversity for overall ecosystem functioning. Ecology, 89(5), 1223–1231. 18543617 10.1890/06-2091.1

[ece310342-bib-0031] Giling, D. P. , Beaumelle, L. , Phillips, H. R. P. , Cesarz, S. , Eisenhauer, N. , Ferlian, O. , Gottschall, F. , Guerra, C. , Hines, J. , Sendek, A. , Siebert, J. , Thakur, M. P. , & Barnes, A. D. (2019). A niche for ecosystem multifunctionality in global change research. Global Change Biology, 25(3), 763–774. 10.1111/gcb.14528 30449061

[ece310342-bib-0032] Grime, J. (1998). Benefits of plant diversity to ecosystems: Immediate, filter and founder effects. Ecology, 86, 902–910.

[ece310342-bib-0033] Hector, A. , & Bagchi, R. (2007). Biodiversity and ecosystem multifunctionality. Nature, 448(7150), 188–190. 10.1038/nature05947 17625564

[ece310342-bib-0034] Hillebrand, H. , Bennett, D. M. , & Cadotte, M. W. (2008). Consequences of dominance: A review of evenness effects on local and regional ecosystem processes. Ecology, 89(6), 1510–1520. 18589516 10.1890/07-1053.1

[ece310342-bib-0035] Huston, M. A. (1997). Hidden treatments in ecological experiments: Re‐evaluating the ecosystem function of biodiversity. Oecologia, 110, 449–460. 28307235 10.1007/s004420050180

[ece310342-bib-0036] Joo, H. S. , Hirai, M. , & Shoda, M. (2005). Characteristics of ammonium removal by heterotrophic nitrification‐aerobic denitrification by *Alcaligenes faecalis* no. 4. Journal of Bioscience and Bioengineering, 100(2), 184–191. 10.1263/jbb.100.184 16198262

[ece310342-bib-0037] Krause‐Jensen, D. , Duarte, C. M. , Hendriks, I. E. , Meire, L. , Blicher, M. E. , Marbà, N. , & Sejr, M. K. (2015). Macroalgae contribute to nested mosaics of pH variability in a subarctic fjord. Biogeosciences, 12(16), 4895–4911. 10.5194/bg-12-4895-2015

[ece310342-bib-0038] Kroeker, K. J. , Kordas, R. L. , Crim, R. , Hendriks, I. E. , Ramajo, L. , Singh, G. S. , Duarte, C. M. , & Gattuso, J. P. (2013). Impacts of ocean acidification on marine organisms: Quantifying sensitivities and interaction with warming. Global Change Biology, 19(6), 1884–1896. 10.1111/gcb.12179 23505245 PMC3664023

[ece310342-bib-0039] Kuznetsova, A. , Brockhoff, P. B. , & Christensen, R. H. B. (2017). lmerTest package: Tests in linear mixed effects models. Journal of Statistical Software, 82(13), 1–26. 10.18637/JSS.V082.I13

[ece310342-bib-0040] Lindeberg, M. R. , & Lindstrom, S. C. (2016). Field guide to seaweeds of Alaska. Alaska Sea Grant. https://seagrant.uaf.edu/bookstore/pubs/SG‐ED‐69.html

[ece310342-bib-0041] Lohbeck, M. , Bongers, F. , Martinez‐Ramos, M. , & Poorter, L. (2016). The importance of biodiversity and dominance for multiple ecosystem functions in a human‐modified tropical landscape. Ecology, 97(10), 2772–2779. 27859119 10.1002/ecy.1499

[ece310342-bib-0042] Losapio, G. , Schmid, B. , Bascompte, J. , Michalet, R. , Cerretti, P. , Germann, C. , Haenni, J. P. , Neumeyer, R. , Ortiz‐Sánchez, F. J. , Pont, A. C. , Rousse, P. , Schmid, J. , Sommaggio, D. , & Schöb, C. (2021). An experimental approach to assessing the impact of ecosystem engineers on biodiversity and ecosystem functions. Ecology, 102(2), e03243. 10.1002/ecy.3243 33190225

[ece310342-bib-0043] Mahanes, S. A. , Bracken, M. E. S. , & Sorte, C. J. B. (2022). Climate change amelioration by marine producers: Does dominance predict impact? Biological Bulletin, 243(3), 299–314. 36716485 10.1086/721229

[ece310342-bib-0044] Mann, K. H. (1973). Seaweeds: Their productivity and strategy for growth. Science, 182, 975–981. 17833778 10.1126/science.182.4116.975

[ece310342-bib-0045] Manning, P. , van der Plas, F. , Soliveres, S. , Allan, E. , Maestre, F. T. , Mace, G. , Whittingham, M. J. , & Fischer, M. (2018). Redefining ecosystem multifunctionality. Nature Ecology and Evolution, 2(3), 427–436. 10.1038/A41559-017-0461-7 29453352

[ece310342-bib-0046] Mantyka‐Pringle, C. S. , Martin, T. G. , & Rhodes, J. R. (2012). Interactions between climate and habitat loss effects on biodiversity: A systematic review and meta‐analysis. Global Change Biology, 18(4), 1239–1252. 10.1111/j.1365-2486.2011.02593.x

[ece310342-bib-0047] Mariotte, P. (2014). Do subordinate species punch above their weight? Evidence from above‐and below‐ground. New Phytologist, 203(1), 16–21. 24635114 10.1111/nph.12789

[ece310342-bib-0048] Mariotte, P. , Robroek, B. J. M. , Jassey, V. E. J. , & Buttler, A. (2015). Subordinate plants mitigate drought effects on soil ecosystem processes by stimulating fungi. Functional Ecology, 29(12), 1578–1586. 10.1111/1365-2435.12467

[ece310342-bib-0049] Moi, D. A. , Evangelista, H. B. A. , Mormul, R. P. , Evangelista, L. R. , & Thomaz, S. M. (2021). Ecosystem multifunctionality and stability are enhanced by macrophyte richness in mesocosms. Aquatic Sciences, 83(3), 53. 10.1007/s00027-021-00808-5

[ece310342-bib-0050] Mouillot, D. , Villéger, S. , Scherer‐Lorenzen, M. , & Mason, N. W. H. (2011). Functional structure of biological communities predicts ecosystem multifunctionality. PLoS One, 6(3), e17476. 10.1371/journal.pone.0017476 21423747 PMC3053366

[ece310342-bib-0051] Noël, L. M. L. J. , Griffin, J. N. , Thompson, R. C. , Hawkins, S. J. , Burrows, M. T. , Crowe, T. P. , & Jenkins, S. R. (2010). Assessment of a field incubation method estimating primary productivity in rockpool communities. Estuarine, Coastal and Shelf Science, 88(1), 153–159. 10.1016/j.ecss.2010.03.005

[ece310342-bib-0052] Orwin, K. H. , Ostle, N. , Wilby, A. , & Bardgett, R. D. (2014). Effects of species evenness and dominant species identity on multiple ecosystem functions in model grassland communities. Oecologia, 174(3), 979–992. 10.1007/s00442-013-2814-5 24213721

[ece310342-bib-0053] Paine, R. T. (1966). Food web complexity and species diversity. The American Naturalist, 100(910), 65–75.

[ece310342-bib-0054] Pfister, C. A. (2007). Intertidal invertebrates locally enhance primary production. Ecology, 88(7), 1647–1653. 17645011 10.1890/06-1913.1

[ece310342-bib-0055] Pfister, C. A. , & Altabet, M. A. (2019). Enhanced microbial nitrogen transformations in association with macrobiota from the rocky intertidal. Biogeosciences, 16(2), 193–206. 10.5194/bg-16-193-2019

[ece310342-bib-0056] R Core Team . (2013). R: A language and environment for statistical computing. R Foundation for Statistical Computing. http://www.r‐project.org/

[ece310342-bib-0057] Semesi, I. S. , Beer, S. , & Björk, M. (2009). Seagrass photosynthesis controls rates of calcification and photosynthesis of calcareous macroalgae in a tropical seagrass meadow. Marine Ecology Progress Series, 382, 41–47. 10.3354/meps07973

[ece310342-bib-0058] Shao, Y. , Wang, X. , Zhao, J. , Wu, J. , Zhang, W. , Neher, D. , Li, Y. , Lou, Y. , & Fu, S. (2016). Subordinate plants sustain the complexity and stability of soil micro‐food webs in natural bamboo forest ecosystems. Journal of Applied Ecology, 53(1), 130–139. 10.1111/1365-2664.12538

[ece310342-bib-0059] Silbiger, N. J. , & Sorte, C. J. B. (2018). Biophysical feedbacks mediate carbonate chemistry in coastal ecosystems across spatiotemporal gradients. Scientific Reports, 8(1), 796. 10.1038/A41598-017-18736-6 29335493 PMC5768679

[ece310342-bib-0060] Slade, E. M. , Kirwan, L. , Bell, T. , Philipson, C. D. , Lewis, O. T. , & Roslin, T. (2017). The importance of species identity and interactions for multifunctionality depends on how ecosystem functions are valued. Ecology, 98(10), 2626–2639. 28722121 10.1002/ecy.1954

[ece310342-bib-0061] Sorte, C. J. B. , & Bracken, M. E. S. (2015). Warming and elevated CO_2_ interact to drive rapid shifts in marine community production. PLoS One, 10(12), e0145191. 10.1371/journal.pone.0145191 26714167 PMC4694712

[ece310342-bib-0062] Tagliarolo, M. , Clavier, J. , Chauvaud, L. , Koken, M. , & Grall, J. (2012). Metabolism in blue mussel: Intertidal and subtidal beds compared. Aquatic Biology, 17(2), 167–180. 10.3354/ab00464

[ece310342-bib-0063] Tingley, M. W. , Orwig, D. A. , Field, R. , & Motzkin, G. (2002). Avian response to removal of a forest dominant: Consequences of hemlock woolly adelgid infestations. Journal of Biogeography, 29, 1505–1516.

[ece310342-bib-0064] Tolkkinen, M. , Mykrä, H. , Markkola, A. M. , Aisala, H. , Vuori, K. M. , Lumme, J. , Pirttilä, A. M. , & Muotka, T. (2013). Decomposer communities in human‐impacted streams: Species dominance rather than richness affects leaf decomposition. Journal of Applied Ecology, 50(5), 1142–1151. 10.1111/1365-2664.12138

[ece310342-bib-0065] Valiente‐Banuet, A. , Aizen, M. A. , Alcántara, J. M. , Arroyo, J. , Cocucci, A. , Galetti, M. , García, M. B. , García, D. , Gómez, J. M. , Jordano, P. , Medel, R. , Navarro, L. , Obeso, J. R. , Oviedo, R. , Ramírez, N. , Rey, P. J. , Traveset, A. , Verdú, M. , & Zamora, R. (2015). Beyond species loss: The extinction of ecological interactions in a changing world. Functional Ecology, 29(3), 299–307. 10.1111/1365-2435.12356

[ece310342-bib-0066] Vanni, M. J. (2002). Nutrient cycling by animals in freshwater ecosystems. Annual Review of Ecology and Systematics, 33, 341–370. 10.1146/annurev.ecolsys.33.010802.150519

[ece310342-bib-0067] Wahl, M. , Schneider Covachã, S. , Saderne, V. , Hiebenthal, C. , Müller, J. D. , Pansch, C. , & Sawall, Y. (2018). Macroalgae may mitigate ocean acidification effects on mussel calcification by increasing pH and its fluctuations. Limnology and Oceanography, 63(1), 3–21. 10.1002/lno.10608

[ece310342-bib-0068] Wohlgemuth, D. , Solan, M. , & Godbold, J. A. (2016). Specific arrangements of species dominance can be more influential than evenness in maintaining ecosystem process and function. Scientific Reports, 6(1), 39325. 10.1038/srep39325 27996034 PMC5171799

[ece310342-bib-0069] Yakovis, E. L. , Artemieva, A. V. , Shunatova, N. N. , & Varfolomeeva, M. A. (2008). Multiple foundation species shape benthic habitat islands. Oecologia, 155(4), 785–795. 10.1007/s00442-007-0945-2 18193291

[ece310342-bib-0070] Zavaleta, E. S. , Pasari, J. R. , Hulvey, K. B. , & Tilman, G. D. (2010). Sustaining multiple ecosystem functions in grassland communities requires higher biodiversity. Proceedings of the National Academy of Sciences of the United States of America, 107(4), 1443–1446. 10.1073/pnas.0906829107 20080690 PMC2824370

