# Supplementary material for: The functional effects of a dominant consumer are altered following the loss of a dominant producer
Source: Ecol Evol. 2023 Aug 2;13(8):e10342. doi: 10.1002/ece3.10342 (PMC10396790; doi:10.1002/ece3.10342)
Supplement: Supplementary file 1 — Appendix S1 [file ECE3-13-e10342-s001.docx]

**Supplemental methods**

*Community surveys*

To measure the abundance of individual species and the overall diversity of the tide pool community, we conducted biodiversity surveys to measure the abundance of species in the pools before and shortly after *N. oregona* removal (Figure A1). During each survey, we pumped water out of each tide pool, set down a flexible mesh quadrat with 10 cm x 10 cm squares at the bottom, measured the surface area occupied by each sessile species (algae and invertebrates; with 10 cm^2^ as the minimum measurement assigned for a species) and counted the mobile invertebrates present (Bracken and Nielsen, 2004; Silbiger and Sorte, 2018). We identified organisms to the lowest taxonomic level possible in the field, which was typically to the species level. Some taxa that could not be identified in the field were grouped together (e.g., “limpets” or “coralline algae”).

*Sample Analysis*

We measured the total alkalinity (TA) of the water samples through open-cell titrations (as in Silbiger and Sorte, 2018) with a T50 titrator and LabX software (Mettler-Toledo AG, Schwerzenbach, Switzerland). We analyzed a certified reference material standard (Marine Physical Laboratory, Scripps Institution of Oceanography, La Jolla, California, USA) at the beginning of each sample analysis session (acceptable range: ±1% error) and adhered to an established protocol for TA analysis (SOP 3b in Dickson et al., 2007; Silbiger and Sorte, 2018).

We analyzed pH for all water samples with a UV-1800 benchtop spectrophotometer (Shimadzu, Carlsbad, California, USA) according to the best practices outlined in Dickson et al. (2007). We divided each water sample into three subsamples and analyzed each separately to maximize precision. We collected initial readings for each subsample at three wavelengths before adding 50 µL of m-cresol dye and re-analyzing each at the same three wavelengths (Liu and Chan, 2010). We used the difference between the initial and dye-added measurements to calculate the pH value of each subsample. We then took the mean of the subsamples with < 0.005 pH unit difference among them (excluding subsamples outside that range) for each water sample to produce a raw pH measurement. We then used CO2calc software (Robbins et al., 2010) to adjust the raw pH value for total alkalinity, temperature, salinity, and stoichiometric dissociation constants and calculate a final pH value on the total scale (Mehrbach et al., 1973; Dickson and Millero, 1987; Kroeker et al., 2021).

We analyzed the frozen 50 mL water samples for dissolved inorganic nutrient concentration (Bracken et al., 2018; Silbiger and Sorte, 2018). We measured NO_3_^-^, NO_2_^-^, and PO_4_^3-^ concentrations (mmol L^-1^) with a QuickChem 8500 Series Analyzer (Lachat Instruments, Loveland, Colorado, USA) and ammonia (NH_4_^+^) concentrations (µmol L^-1^) with the phenolhypochlorite method (Solórzano, 1969) on a UV-1800 benchtop spectrophotometer (Shimadzu, Carlsbad, California, USA).
